# Longitudinal Clinical Profiles of Hospital vs. Community-Acquired Acute Kidney Injury in COVID-19

**DOI:** 10.3389/fmed.2021.647023

**Published:** 2021-05-28

**Authors:** Justin Y. Lu, Ioannis Babatsikos, Molly C. Fisher, Wei Hou, Tim Q. Duong

**Affiliations:** ^1^Department of Radiology, Montefiore Medical Center, Albert Einstein College of Medicine, New York, NY, United States; ^2^Renaissance School of Medicine, Stony Brook University, Stony Brook, NY, United States; ^3^Division of Nephrology, Department of Medicine, Montefiore Medical Center, Albert Einstein College of Medicine, New York, NY, United States; ^4^Department of Family, Population & Preventive Medicine, Stony Brook Medicine, New York, NY, United States

**Keywords:** SARS-CoV-2, AKI, d-dimer, lactate dehydrogenase, multiorgan failure, cytokine storm, kidney disease

## Abstract

Acute kidney injury (AKI) is associated with high mortality in coronavirus disease 2019 (COVID-19). However, it is unclear whether patients with COVID-19 with hospital-acquired AKI (HA-AKI) and community-acquired AKI (CA-AKI) differ in disease course and outcomes. This study investigated the clinical profiles of HA-AKI, CA-AKI, and no AKI in patients with COVID-19 at a large tertiary care hospital in the New York City area. The incidence of HA-AKI was 23.26%, and CA-AKI was 22.28%. Patients who developed HA-AKI were older and had more comorbidities compared to those with CA-AKI and those with no AKI (*p* < 0.05). A higher prevalence of coronary artery disease, heart failure, and chronic kidney disease was observed in those with HA-AKI compared to those with CA-AKI (*p* < 0.05). Patients with CA-AKI received more invasive and non-invasive mechanical ventilation, anticoagulants, and steroids compared to those with HA-AKI (*p* < 0.05), but patients with HA-AKI had significantly higher mortality compared to those with CA-AKI after adjusting for demographics and clinical comorbidities (adjusted odds ratio = 1.61, 95% confidence interval = 1.1–2.35, *p* < 0.014). In addition, those with HA-AKI had higher markers of inflammation and more liver injury (*p* < 0.05) compared to those with CA-AKI. These results suggest that HA-AKI is likely part of systemic multiorgan damage and that kidney injury contributes to worse outcomes. These findings provide insights that could lead to better management of COVID-19 patients in time-sensitive and potentially resource-constrained environments.

## Introduction

Coronavirus disease 2019 (COVID-19) (1, 2) caused by the novel severe acute respiratory syndrome coronavirus (SARS-CoV-2) is the worst public health disaster of the century, accounting for millions of infections and deaths globally. Hospital-acquired acute kidney injury (HA-AKI) (3–15) is a frequent complication of COVID-19 that is associated with critical illness and increased mortality. Proposed mechanisms of AKI in patients with COVID-19 include volume depletion, hypotension, sepsis, cytokine storm, and direct viral-induced AKI, although the latter has not been proven (16).

Most studies on AKI associated with COVID-19 have investigated clinical variables upon hospital admission as predictors of AKI. However, patients are hospitalized at different stages of disease severity, and some patients present with AKI upon admission, known as community-acquired AKI (CA-AKI). It is unclear whether HA-AKI and CA-AKI COVID-19 patients differ in disease severity, course, and outcomes. There are currently limited data on differences in the clinical characteristics and outcomes among patients with HA-AKI and CA-AKI who are hospitalized with COVID-19. A better understanding of the clinical presentation and outcomes of HA-AKI and CA-AKI in the context of COVID-19 may allow for earlier recognition, intervention, and improvement in clinical outcomes.

The purpose of our study was to investigate the temporal characteristics of clinical variables and mortality rate among patients hospitalized with COVID-19 with HA-AKI, CA-AKI, and no AKI. We compared the temporal progression of clinical variables (with time lock to HA-AKI onset), demographics, comorbidities, escalated care, in-hospital mortality, and association with treatments [invasive mechanical ventilation (IMV), non-invasive ventilation, anticoagulants, and steroids] between HA-AKI and CA-AKI COVID-19 patients.

## Methods

### Study Population and Data Collection

**Figure 1:**
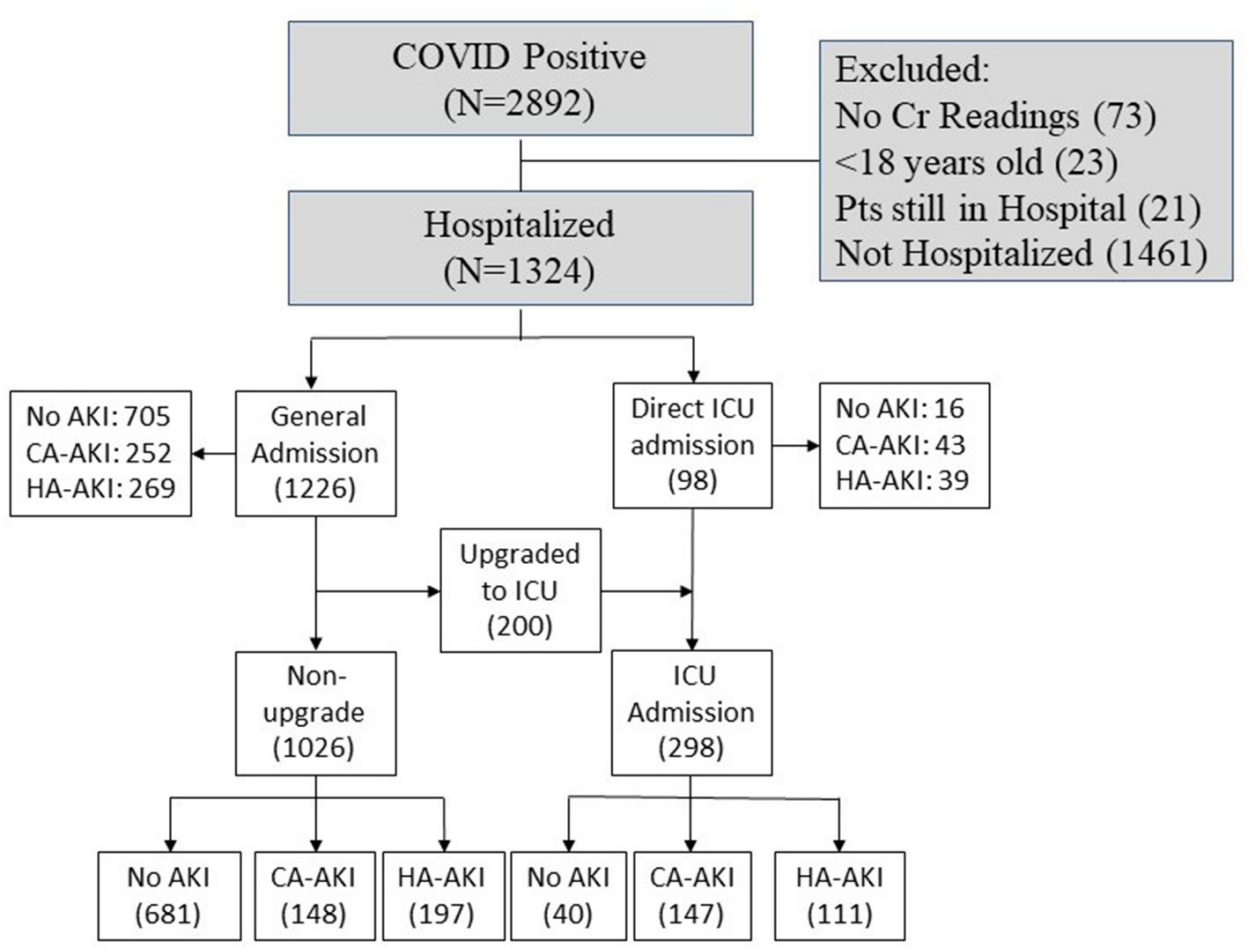
Flowchart of patient selection. Pts, patients; ICU, intensive care unit; Cr, creatinine.

This retrospective study was approved by Stony Brook University Institutional Research Board with exemption of informed consent. A subset of these data has been used to study different aspects of COVID-19 disease (17–28). Figure 1 shows patients who presented to the emergency department (ED) and were hospitalized with COVID-19 from February 7, 2020, to June 30, 2020. There were 2,892 patients who tested positive using real-time polymerase chain reaction test for SARS-CoV-2 on a nasopharyngeal swab specimen. Following exclusion of non-hospitalized patients, patients < 18 years old, those missing creatinine (Cr) data, those with end-stage kidney disease, and patients still hospitalized at the time of analysis, there were 1,324 COVID-19–positive patients analyzed.

AKI was defined using the Kidney Disease Improving Global Outcomes (KDIGO) criteria (29, 30) as either a 0.3-mg/dL increase in serum Cr within 48 h or a 1.5 × increase in serum Cr. The lowest Cr value during hospitalization was used as the baseline Cr due to lack of data prior to hospitalization (31, 32). CA-AKI was defined as AKI within 24 h of admission. Urine output was not used to define AKI as it was not reliably documented. AKI was staged per KDIGO guidelines: stage 1: ≥0.3 mg/dL or to >1.5 to 2 × increase in Cr; stage 2: >2 to ≤ 3 × increase in Cr; and stage 3: increase >3 × increase in Cr or rise to ≥4.0 mg/dL or new initiation of renal replacement therapy (RRT) (29, 30).

Demographics, clinical comorbidities, longitudinal vital signs, laboratory blood tests, and blood gases were extracted from electronic medical records. Demographic data included age, sex, ethnicity, and race. Chronic comorbidities included smoking, diabetes, hypertension, asthma, chronic obstructive pulmonary disease (COPD), coronary artery disease, heart failure, cancer, immunosuppression, and chronic kidney disease. Longitudinal laboratory tests included Cr, procalcitonin (procal), aspartate transaminase (AST), alanine aminotransferase (ALT), ferritin (ferr), lactate dehydrogenase (LDH), white blood cell (WBC) count, C-reactive protein (CRP), lymphocyte count, d-dimer (ddim), brain natriuretic peptide (BNP), and albumin. Longitudinal vital signs included heart rate (HR), diastolic blood pressure (DBP), systolic blood pressure (SBP), respiratory rate (RR), pulse oxygen saturation (Spo_2_), and temperature. Longitudinal blood gas variables include po_2_, pco_2_, and pH.

Laboratory test variables were plotted across time with time lock (*t* = 0) to AKI onset, along with data 3 days before and 3 days after AKI onset. For comparison, time-series data for no-AKI patients were time locked (*t* = 0) to 3 days after ED admission, along with data 3 days before and 3 days after that time point. The admission at 3 days post-ED was chosen because HA-AKI COVID-19 patients developed AKI 3.3 days, on average, after ED admission (see *Results*). The mean of HA-AKI onset was taken with exclusion of day 0 because patients with AKI within 24 h were considered as CA-AKI. For ease of comparison, individual laboratory test values were normalized to the no-AKI group at *t* = 0 for individual patients. For the CA-AKI cohort, laboratory values were plotted with *t* = 0, indicating the day of hospital admission.

In addition, we also extracted data on IMV, non-invasive mechanical ventilation, prophylactic and therapeutic anticoagulants, steroid, and RRT.

### Statistical Analysis

All statistical analyses were performed using SAS software. Frequencies and percentages for categorical variables between HA-AKI and CA-AKI, between HA-AKI and no AKI, and between CA-AKI and no-AKI groups were compared using χ^2^ tests. Continuous variables, expressed as median (interquartile range), were compared between groups using non-parametric Kruskal–Wallis test. Mortality rates were compared between groups with χ^2^ tests adjusted with covariates. Differences between HA-AKI and CA-AKI group for clinical variables in time-series graphs were analyzed via linear mixed model and least-squares means test with Tukey adjustment. *P* < 0.05 was considered statistically significant unless otherwise specified. *p* values and odds ratios (ORs) for mortality were calculated using logistic regression adjusted for age, ethnicity, and comorbidities that were significantly different between groups for the perspective groups.

## Results

### Baseline Characteristics

Table 1 summarizes patient demographics and comorbidities of the HA-KI, CA-AKI, and no-AKI groups. HA-AKI patients were older than CA-AKI patients and no-AKI patients (*p* < 0.05). There were 148 CA-AKI, 197 HA-AKI, and 681 no-AKI patients admitted to the general floor. In the intensive care unit (ICU) or upgraded-to-ICU cohort (*n* = 298), there were 147 CA-AKI, 111 HA-AKI, and 40 no-AKI patients. There was no significant difference between sex or race between the groups. There was significantly less AKI observed in patients of Hispanic ethnicity compared to those with no AKI (*p* < 0.05). HA-AKI had significantly higher prevalence of smoking, diabetes, hypertension, coronary artery disease, cancer, and chronic kidney disease (*p* < 0.05), but not asthma, COPD, and immunosuppression (*p* > 0.05), compared to no AKI. CA-AKI had significantly higher prevalence of smoking, diabetes, hypertension, cancer, immunosuppression, and chronic kidney disease (*p* < 0.05), but not asthma, COPD, coronary artery disease, and heart failure (*p* > 0.05), compared to no AKI. HA-AKI had significantly higher prevalence of coronary artery disease, heart failure, and chronic kidney disease (*p* < 0.05), but not hypertension, diabetes, smoking, cancer, immunosuppression, asthma, and COPD (*p* > 0.05), compared to CA-AKI. Overall, HA-AKI patients generally had the most comorbidities, followed by CA-AKI and no-AKI patients.

**Table 1:** Demographic characteristics and comorbidities of HA-AKI, CA-AKI, and no-AKI patients.

	**Patients, no. (%)**			
	**HA-AKI**	**CA-AKI**	**No AKI**	***p***
	**(*n* = 308)**	**(*n* = 295)**	**(*n* = 721)**	
**Demographics**
Age, median (range), y	70 (56, 80)	65 (53, 75)	59 (47, 73)	a, b, c
Sex
Male	190 (61.7%)	166 (56.3%)	409 (56.7%)	
Female	118 (38.3%)	129 (43.7%)	312 (43.3%)	
Ethnicity				b
Hispanic/Latino	62 (20.1%)	74 (25.1%)	197 (27.3%)	
Non-Hispanic/Latino	214 (69.5%)	180 (61.0%)	419 (58.1%)	
Unknown	32 (10.4%)	41 (13.9%)	105 (14.6%)	
Race				
Caucasian	177 (57.5%)	165 (55.9%)	384 (53.3%)	
African American	26 (8.4%)	13 (4.4%)	52 (7.2%)	
Asian	10 (3.3%)	15 (5.1%)	22 (3.1%)	
American Indian/Alaska Native	1 (0.3%)	2 (0.7%)	1 (0.1%)	
Native Hawaiian or other Pacific Islander	0	0	1 (0.1%)	
More than one race	1 (0.3%)	2 (0.7%)	4 (0.6%)	
Unknown/not reported	93 (30.2%)	98 (33.2%)	257 (35.6%)	
**Comorbidities**
Smoking history				b, c
Current smoker	18 (5.8%)	12 (4.1%)	27 (3.7%)	
Former smoker	77 (25.0%)	61 (20.7%)	160 (22.2%)	
Never smoked	189 (61.4%)	193 (65.4%)	505 (70.0%)	
Unknown	24 (7.8%)	29 (9.8%)	29 (4.1%)	
Diabetes	108 (35.1%)	97 (32.9%)	159 (22.1%)	b, c
Hypertension	196 (63.6%)	175 (59.3%)	302 (41.9%)	b, c
Asthma	21 (6.8%)	18 (6.1%)	46 (6.4%)	
COPD	33 (10.7%)	32 (10.9%)	57 (7.9%)	
Coronary artery disease	73 (23.7%)	44 (14.9%)	91 (12.6%)	a, b
Heart failure	55 (17.9%)	27 (9.2%)	38 (5.3%)	a, b
Cancer	40 (13.0%)	37 (12.6%)	59 (8.2%)	b, c
Immunosuppression	30 (9.7%)	29 (9.8%)	47 (6.5%)	c
Chronic kidney disease	95 (18.8%)	37 (12.5%)	45 (6.2%)	a, b, c
No. of comorbidities of each patient				a, b, c
0	57 (18.5%)	82 (27.8%)	275 (38.1%)	
1	71 (23.1%)	55 (18.6%)	205 (28.4%)	
2	70 (22.7%)	87 (29.5%)	136 (18.9%)	
3	60 (19.5%)	36 (12.2%)	69 (9.6%)	
4	31 (10.0%)	20 (6.8%)	26 (3.6%)	
5	15 (4.9%)	11 (3.7%)	5 (0.7%)	
6	4 (1.3%)	4 (1.4%)	4 (0.6%)	
7	0	0	1 (0.1%)	

*Group comparison of categorical variables in frequencies and percentages used χ^2^ test or Fisher exact tests. Group comparison of continuous variables in medians and interquartile ranges (IQR) used the Mann–Whitney U test. p < 0.05 for ^a^between CA-AKI and HA-AKI, ^b^between HA-AKI and no AKI, and ^c^between CA-AKI and No AKI*.

*COPD, chronic obstructive pulmonary disease*.

### Time Course of AKI

**Figure 2:**
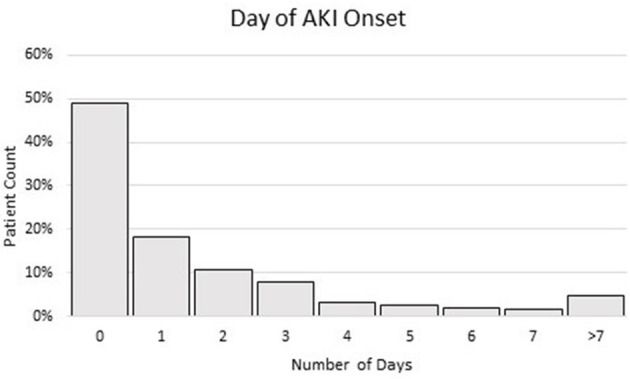
Histogram of % of patients when AKI was developed in days after hospitalization. Patient having AKI at day 0 (within 24 h of ED admission) was taken as indication of CA-AKI. Patient having AKI on day 1 and after was taken as HA-AKI. The total sample size was 603 AKI patients.

The time course of when patients developed AKI upon and following hospitalization is shown in Figure 2. Approximately half of the AKI patients (48.9%) presented with AKI on the day of hospital admission, which was taken as CA-AKI. Following hospitalization, the incidence of AKI decreased over time. Among hospitalized patients with COVID-19, 18.2, 10.8, 7.8, 3.3, 2.7, 1.8, 1.7, and 4.8% developed AKI after 1, 2, 3, 4, 5, 6, 7, and >7 days following hospitalization, respectively. HA-AKI patients developed AKI, on average, after 3.3 days of hospitalization [this value excluded day 0 (CA-AKI)].

### Temporal Characteristics of Laboratory Variables

**Figure 3:**
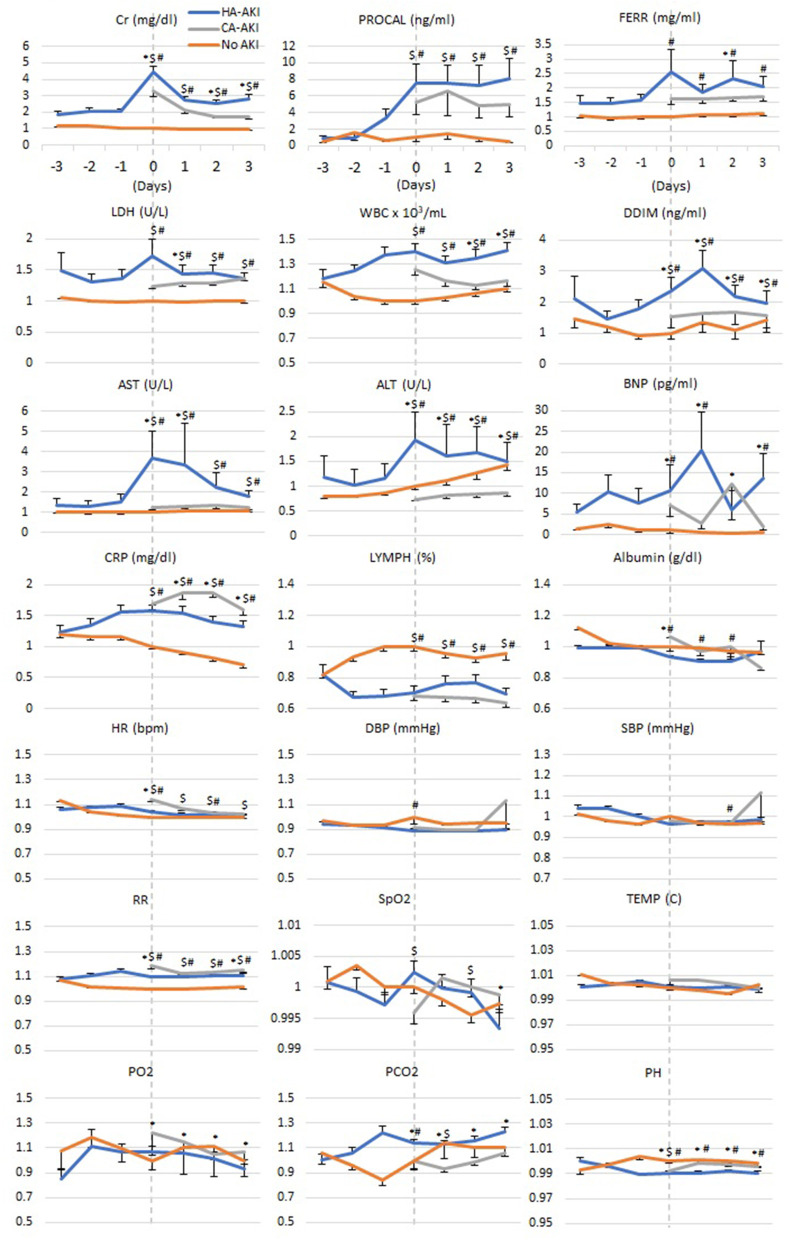
Temporal progression of laboratory tests, vital signs, and blood gases with *t* = 0 representing day of AKI onset in HA-AKI patients. No-AKI patient data were centered around the third day after hospital admission. For CA-AKI, *t* = 0 was taken as the day of hospital admission when Cr was already elevated. No AKI, non-AKI; CA-AKI, community-acquired AKI; HA-AKI, hospital-acquired AKI. Values are normalized by dividing all data points by value of reading at time 0 of the no-AKI group. Cr, creatinine; BNP, brain natriuretic peptide; ALT, Alanine Aminotransferase; AST, aspartate aminotransferase; PROCAL, procalcitonin; CRP, C-reactive protein; LDH, lactate dehydrogenase; WBC, white blood cell; DBP, diastolic blood pressure; SBP, systolic blood pressure. Error bars are SEM. *p < 0.05 between HA-AKI and CA-AKI, ^#^*p* < 0.05 between HA-AKI and no AKI, ^$^*p* < 0.05 between CA-AKI and no AKI.

Figure 3 depicts the time series of laboratory tests relative to AKI onset for HA-AKI, CA-AKI, and no-AKI cohorts. For ease of comparison, these laboratory values were normalized to the no-AKI group at *t* = 0 by individual patients. No AKI Cr remained low and time invariant; HA-AKI Cr spiked 4.5 times of baseline at *t* = 0 and returned toward baseline but above no AKI level, and CA-AKI Cr was 3.1 times higher than no AKI at *t* = 0 but was not nearly as high as HA-AKI patients.

When comparing HA-AKI and no-AKI groups, procal was elevated 1 day prior to AKI onset, peaked the same time as Cr, and remained elevated. CRP, WBC, ddim, BNP, and lymphocyte showed divergence between HA-AKI and those with no AKI starting 2 days prior to development of AKI. Lymphocyte counts of HA-AKI and CA-AKI were lower compared to patients with no AKI. AST, ALT, ferr, and LDH showed similar temporal patterns as Cr. Most vital signs and blood gases (HR, DBP pressure, SBP, Spo_2_, temperature, po_2_, and pco_2_) and albumin were similar between groups across time, except that RR was elevated, and pH was lower in the HA-AKI group compared to the no-AKI group.

In those with CA-AKI, Cr, Procal, LDH, WBC, and ddim levels were between those of HA-AKI and no-AKI groups. AST, ALT, and BNP of CA-AKI were closer to those of no AKI. CRP of CA-AKI was above that of HA-AKI. Lymph, albumin, and most vital signs and blood gases (HR, RR, DBP pressure, SBP, Spo_2_, temperature, po_2_, and pco_2_, pH) of CA-AKI were similar to those of HA-AKI. Markers of inflammation were higher in those with AKI compared to those without AKI. However, those with CA-AKI had lower levels of these markers compared to those with HA-AKI.

### Treatments Effects

We also investigated the differences in treatments patients received (Table 2). CA-AKI patients generally received significantly more invasive and non-invasive mechanical ventilation, prophylactic and therapeutic anticoagulants, and steroids compared to HA-AKI patients (*p* < 0.05). Both HA-AKI and CA-AKI received more IMV, non-invasive mechanical ventilation, prophylactic and therapeutic anticoagulants, steroid, and RRT compared to those without AKI (*p* < 0.05).

**Table 2:** Treatments utilized for HA-AKI, CA-AKI, and no-AKI groups.

	**Patients, no. (%)**			
	**HA-AKI**	**CA-AKI**	**No AKI**	***p***
	**(*n* = 308)**	**(*n* = 295)**	**(*n* = 721)**	
Invasive mechanical ventilation	99 (32.1%)	114 (38.6%)	17 (2.4%)	b, c
Noninvasive mechanical ventilation	37 (12.0%)	59 (20.0%)	30 (4.2%)	a, b, c
**Anticoagulants**				
Prophylactic	153 (49.7%)	172 (58.3%)	212 (29.4%)	a, b, c
Therapeutic	86 (27.9%)	87 (29.5%)	59 (8.2%)	b, c
Steroids	99 (32.1%)	119 (40.3%)	69 (9.6%)	a, b, c
**Renal replacement therapy**				
Continuous renal replacement therapy	5 (1.6%)	5 (1.7%)	0	ns
Hemodialysis	29 (9.4%)	22 (7.5%)	0	ns
Both continuous renal replacement therapy and hemodialysis	16 (5.2%)	2 (0.7%)	0	a

*Group comparison of categorical variables in frequencies and percentages used χ^2^ test or Fisher exact tests. p < 0.05 for ^a^between CA-AKI and HA-AKI, ^b^between HA-AKI and no AKI, and ^c^between CA-AKI and no AKI. ns, not significant between any pairs*.

### In-hospital Mortality

Table 3A describes the incidence of AKI, admission status, and in-hospital mortality between the groups. Of the hospitalized COVID-19 patients (*n* = 1,324), the total incidence of AKI was 43.54%. Of the patients with AKI, there was a similar incidence of HA-AKI (23.26%) and CA-AKI (22.28%) that was not statistically significant. More patients without AKI were admitted to a general medical floor and remained on a general medical floor, and fewer were directly admitted or upgraded to ICU compared to HA-AKI and CA-AKI (*p* < 0.05 pairwise comparison). Notably, there were more CA-AKI patients upgraded to ICU compared to HA-AKI, and fewer CA-AKI patients remained in general floor compared to HA-AKI (*p* < 0.05).

**Table 3:** **(A)** Numbers of patients with HA-AKI, CA-AKI, and no AKI and their mortality rates separated by general floor and ICU admission, and **(B)** adjusted mortality odds ratios for different groups.

	**Patients, no. (%)**			
	**HA-AKI**	**CA-AKI**	**No AKI**	***p***
**(A)**				
**Patient count**				
All admitted patients	308 (23.3%)	295 (22.3%)	721 (54.5%)	b, c
General floor	269 (21.9%)	252 (20.6%)	705 (57.5%)	b, c
Direct ICU admission	39 (39.8%)	43 (43.9%)	16 (16.3%)	b, c
ICU upgrade	72 (36%)	104 (52%)	24 (12%)	a, b, c
Non-upgrade general floor	197 (19.2%)	148 (14.4%)	681 (66.4%)	a, b, c
Total ICU admission	111 (37.2%)	147 (49.3%)	40 (13.4%)	a, b, c
**Mortality**				
All admitted patients	31.5	21.0	6.9	a, b, c
General floor	26.40	18.30	6.50	a, b, c
Direct ICU admission	66.70	37.20	25	a, b
ICU upgrade	58.30	30.80	20.80	a, b
Non-upgrade general floor	14.70	9.50	6.00	b
Total ICU admission	61.30	32.70	22.50	a, b
**Adjusted mortality**	**Adjusted OR [95% CI]**		**Adjusted** ***p***	
**(B)**				
HA-AKI and no AKI	4.67 [3.1, 7.0]		< 0.001	
HA-AKI and CA-AKI	1.61 [1.10, 2.35]		0.014	
CA-AKI and no AKI	3.39 [2.22, 5.21]		< 0.001	

*ORs and p values were adjusted for covariates (see Methods)*.

*p < 0.05 for ^a^between CA-AKI and HA-AKI, ^b^between HA-AKI and no AKI, and ^c^between CA-AKI and no AKI*.

Of all hospitalized COVID-19 patients, in-hospital mortality was highest in the HA-AKI cohort (31.5%), followed by CA-AKI (21%) and then the group with no AKI (6.9%) (*p* < 0.05 pairwise comparison). Mortality in patients who developed HA-AKI was 1.5–1.9 times higher compared to patients with CA-AKI and 2.5–4.1 times higher compared to those with no AKI (unadjusted). After adjusting for age and all significant comorbidities, HA-AKI had higher mortality compared to patients with CA-AKI [adjusted OR = 1.61, 95% confidence interval (CI) = 1.1–2.35, *p* < 0.014] and those with no AKI (adjusted OR = 4.67, 95% CI = 3.1–7.0, *p* < 0.001), and patients with CA-AKI had higher mortality than those with no AKI (adjusted OR = 3.39, 95% CI = 2.2–5.21, *p* < 0.001) (Table 3B). We also evaluated the mortality by AKI severity using KDIGO staging. The compositions between HA-AKI and CA-AKI across different stages of AKI were similar. Mortality was highest for stage 3 AKI in both patients with HA-AKI and CA-AKI (Table 4).

**Table 4:** Composition and (unadjusted) mortality of HA-AKI and CA-AKI for different stages of kidney disease.

	**Composition**		**Mortality**		
	**HA-AKI**	**CA-AKI**	**HA-AKI**	**CA-AKI**	***p***
	**(*n* = 308)**	**(*n* = 295)**	**(*n* = 308)**	**(*n* = 295)**	
Stage 1	145 (47.1%)	113 (38.3%)	28 (19.3%)	14 (12.4%)	0.135
Stage 2	57 (18.5%)	73 (24.7%)	19 (33.3%)	8 (11.0%)	**0.002**
Stage 3	106 (34.4%)	109 (37.0%)	50 (47.2%)	40 (36.7%)	0.120

*p values indicate difference between HA-AKI and CA-AKI for mortality. Bold values indicate a significant p value*.

## Discussion

We evaluated the temporal characteristics of clinical variables and mortality in patients hospitalized with COVID-19 with HA-AKI, CA-AKI, and no AKI. We found that patients who developed HA-AKI were older and had more comorbidities and a higher prevalence of coronary artery disease, heart failure, and chronic kidney disease compared to patients with CA-AKI. Patients with HA-AKI and CA-AKI more often required ICU level of care and had higher mortality compared to those without AKI. Although CA-AKI patients more often required invasive and non-invasive ventilation, patients with HA-AKI had the highest mortality. Patients with HA-AKI also had higher markers of inflammation, thrombosis, and cell death compared to patients with CA-AKI. To our knowledge, this is the first study to compare differences in clinical profiles between HA-AKI and CA-AKI COVID-19 patients.

While there are many studies investigating AKI incidence in COVID-19 patients, few studies have evaluated differences between HA-AKI and CA-AKI COVID-19 patients. Hirsch et al. reported CA-AKI incidence of 37.3% in COVID-19 patients in a major New York City hospital system (31), compared to an incidence of 22.28% in our study. They also defined CA-AKI as development of AKI within 24 h of hospital admission. In contrast, Pelayo et al. reported a significantly higher CA-AKI incidence of 72% among patients hospitalized with COVID-19 in a tertiary inner-city hospital in Philadelphia in 110 COVID-19 patients (32). It is possible these wide variations in the incidence of CA-AKI are due to differences in the definition of CA-AKI or heterogeneous patient populations.

HA-AKI patients were significantly older compared to CA-AKI patients and those with no AKI. Although other studies have identified an association between black race and AKI in the context of COVID-19 (10, 33, 34), we were unable to determine if race was associated with AKI as our patient population was predominantly Caucasian or of unknown race. Patients with HA-AKI had a higher number of comorbidities and higher prevalence of coronary artery disease, heart failure, and chronic kidney disease compared to patients with CA-AKI. These findings are consistent with a prior report that identified these comorbidities as risk factors for HA-AKI in patients with COVID-19 (31). This suggests that patients with these comorbidities hospitalized with COVID-19 are at increased risk of development of in-hospital AKI and underscores the importance of close monitoring and early intervention in this high-risk patient population.

Our ICU admission and mortality rate of patients with AKI are similar to previous studies (9–15). We found that the severity of AKI among patients with CA-AKI was similar to patients with HA-AKI. Interestingly, CA-AKI patients received more escalated care and other interventions including invasive and non-invasive mechanical ventilation, prophylactic and therapeutic anticoagulants, and steroids compared to those with HA-AKI. However, patients with HA-AKI were more likely to experience in-hospital mortality. Although it is unclear why patients with CA-AKI had superior short-term outcomes compared to those with HA-AKI, it is possible that these differences may be explained by earlier recognition, referral to nephrology, and appropriate management of CA-AKI. Another possible explanation for differences observed in short-term survival between these groups includes differences in the underlying pathophysiology of AKI. Our findings are consistent with Pelayo et al. who found >2-fold higher mortality among hospitalized patients with COVID-19 who developed HA-AKI compared to those with CA-AKI (32), as well as a recent meta-analysis of non–COVID-19 patients who demonstrated a 2.33-fold higher odds of mortality in patients with HA-AKI compared to those with CA-AKI (35).

Randomized trials have demonstrated that patients hospitalized with COVID-19 who require supplemental oxygen who are treated with steroids have improved short-term survival. A recent *post hoc* analysis of a placebo-controlled, randomized trial (36) suggests that intraoperative steroids might lower the risk of AKI requiring RRT after cardiac surgery (37). In our study, patients with CA-AKI were more likely to receive steroids compared to patients with HA-AKI, which may explain the lower in-hospital mortality observed in this group.

Compared to those with HA-AKI, we found those with CA-AKI had lower markers of inflammation, cell death, and thrombosis. In addition, patients with CA-AKI showed little or no elevation in AST and ALT, indicators of absence of liver injury compared to those with HA-AKI. These findings suggest that the same or similar factors (cytokine storm) might have caused AKI and liver injury in patients with HA-AKI, and the mechanisms responsible for AKI in patients with CA-AKI may differ compared to those with HA-AKI. Many of the clinical variables in patients with HA-AKI and CA-AKI were markedly worse compared to those with no AKI and fluctuated temporally to a greater extent, highlighting that patients with HA-AKI and CA-AKI had more severe COVID-19. Taken together, these findings suggest that AKI is a result of multiorgan failure contributing to higher mortality rate. Time-locked longitudinal data can provide insight into AKI progression that may lead to earlier recognition of AKI, intervention, and improvement in clinical outcomes.

Strengths of our study include our large sample size and use of longitudinal variables to characterize disease course among patients hospitalized with COVID-19 who developed AKI. As with any observational study, ours has limitations. We did not have urinalysis data, and both proteinuria and hematuria are associated with AKI and in-hospital mortality. Future studies should include urinalysis data in models to predict AKI. In addition, we did not have a baseline Cr preceding hospitalization, which may have led to an overestimation of CA-AKI. However, we compared the nadir Cr value during the hospitalization to retroactively define and stage AKI, as done by Siew and Matheny (38). Finally, this was a single-center study that investigated only short-term outcomes following AKI. Future studies are needed to determine long-term outcomes such as development of CKD, dependence on RRT, and mortality following HA-AKI and CA-AKI among patients with COVID-19.

## Conclusions

This study compared the clinical profiles between COVID-19 patients with HA-AKI and CA-AKI with respect to temporal progression of clinical variables, demographics, comorbidities, escalated care, mortality, and association with treatments. The temporal progression of the laboratory profiles suggests that patients with HA-AKI and CA-AKI may have different manifestations of COVID-19. Our findings suggest that HA-AKI is likely part of the multiorgan failure, has a more severe course than CA-AKI, and that kidney injury contributes but may not be the main cause of worse outcomes. These findings may contribute to improved management of patients with COVID-19 who develop AKI.

## Data Availability Statement

Requests to access these datasets should be directed to Tim Q. Duong, Tim.duong@einsteinmed.org.

## Ethics Statement

The studies involving human participants were reviewed and approved by Stony Brook University IRB-2020-00207. Written informed consent for participation was not required for this study in accordance with the national legislation and the institutional requirements.

## Author Contributions

JL and WH analyzed and edited paper. IB and MF drafted and edited paper. TD designed, supervised, wrote paper, and approved final version. All authors contributed to the article and approved the submitted version.

## Conflict of Interest

The authors declare that the research was conducted in the absence of any commercial or financial relationships that could be construed as a potential conflict of interest.
